# Spatial control of chirality in supramolecular aggregates

**DOI:** 10.1038/srep44094

**Published:** 2017-03-09

**Authors:** Maria A. Castriciano, Denis Gentili, Andrea Romeo, Massimiliano Cavallini, Luigi Monsù Scolaro

**Affiliations:** 1 Consiglio Nazionale delle Ricerche, Istituto per lo Studio dei Materiali Nanostrutturati, Italy; 2 Dipartimento di Scienze Chimiche, Biologiche, Farmaceutiche ed Ambientali, University of Messina, and C.I.R.C.M.S.B., V.le F. Stagno D’Alcontres 31, Vill. S. Agata, 98166 Messina, Italy

## Abstract

Chirality is one of the most intriguing properties of matter related to a molecule’s lack of mirror symmetry. The transmission of chirality from the molecular level up to the macroscopic scale has major implications in life sciences but it is also relevant for many chemical applications ranging from catalysis to spintronic. These technological applications require an accurate control of morphology, homogeneity and chiral handedness of thin films and nanostructures. We demonstrate a simple approach to specifically transfer chirality to the model supramolecular system of J aggregates of the protonated form of tetrakis(4-sulfonatophenyl)-porphyrin by utilizing a soft lithography technique. This approach successfully allows the fabrication of an ordered distribution of sub-micrometric structures in precise and controllable positions with programmed chirality, providing a fundamental breakthrough toward the exploitation of chiral supramolecular aggregates in technological applications, such as sensors, non-linear optics and spintronic.

Since the early resolution experiments in 1848 by Louis Pasteur and the later definition by Lord Kelvin in 1894^1^, chirality has always been one of the hottest topics in chemistry and material science due to its manifold implication in life science, pharmaceutics and catalysis. Chirality occurs at the molecular level as enantiomers of an asymmetric carbon centred molecule and at the supramolecular level, on different length-scales^2^, from asymmetric structural arrangements, such as helices. In the last decades, chirality has been extended to applications in non-linear optics^3^ and more recently in spintronics^4^,^5^. These applications aim to combine the specific optical properties of chirality with the advantage of a supramolecular approach. However, their development is hindered by many problems, which appear transferring chiral systems from solution to solid state, since technological applications require accurate control of morphology, homogeneity and a controlled chiral handedness of thin films and nanostructures. To date, a strong effort has been focused on growing spatially uniform chiral films with a programmed handedness^6^, supramolecular structures^7^, in the artificial fabrication of chiral nanostructures^8^ and in patterning^9^,^10^. However, the local spatial control of handedness of chiral aggregates is not demonstrated and it is still a crucial open challenge.

Here, we successfully address this problem in a model system, by using a straightforward procedure based on patterning, which combines deposition in confinement with the local induction of specific chirality imprinted by a chiral templating agent. In particular, we drive the self-assembly of a model porphyrin into chiral aggregates on a substrate by a wet lithographic method in which a polymeric stamp is functionalised by the two enantiomeric forms of an organic chiral acid, exploiting the so named “sergeants and soldiers effect”^11^ under lithographic control.

J-aggregates of porphyrins are largely studied as model systems^12^,^13^,^14^,^15^,^16^,^17^,^18^,^19^,^20^,^21^ due to their peculiar optical properties; e.g., large extinction coefficients, resonant light scattering^16^, light harvesting^17^ and giant third-order optical nonlinearities^12^. Chirality in these systems adds the possibility of interaction with circular polarized light and their investigation by circular dichroism. Depending on the protocol for their preparation, porphyrin J-aggregates exhibit a large variety of morphologies, in which the molecular building blocks can self-organize into intrinsically chiral units^13^,^14^, giving racemates (i.e. mixtures of left-handed and right-handed chiral units) that can be easily biased through a variety of physical perturbations, such as vortex^18^, rotational and magnetic forces^22^, or using chemical chiral templates^19^. Kinetics of the assembling process control the eventual manifestation of chirality, both in bulk solution^20^ and in confined environments, such as in microemulsions^20^, or microfluidic conditions^21^.

We have proven our approach using tetrakis(4-sulfonatophenyl)-porphyrin (H_2_TPPS_4_^4−,^ (**1**)), which we consider as a representative for this class of nanoaggregates. The protonated green form of **1** (H_4_TPPS_4_^2−^) self-organizes into J-aggregates^13^,^14^, whose formation can be easily detected by the large shift of the B-band to lower energy (<25 eV), by its narrowing and the occurrence of intense resonant light scattering effects^16^. This self-assembly process is highly hierarchical, and is driven by concentration, pH, ionic strength and/or presence of cationic species^23^. Chirality can be induced by chiral templating reagents into J-aggregates supramolecular assemblies that exhibit intense circular dichroism signals related to the specific handedness of the template^19^,^20^. Characterization of the supramolecular aggregates in solution is reported in SI.

Here we prove our procedure by fabricating a few tens of nanometers thick, ordered distribution of sub-micrometric structures in a precise and controllable position that display programmed chirality. As a printing method we used lithographically controlled wetting (LCW)^24^ which is a known technique already used to pattern functional materials, exploiting their self-organizing properties in confinement^25^,^26^,^27^. LCW is an effective and efficient tool to transfer the conditions developed in solution, including the experimental conditions used to drive the aggregation and with minor adjustments, to nanopatterning, even when we tested similar procedures by means of other soft-lithographic methods^28^, including micromolding in capillary^29^, micro transfer molding^30^ and solvent assisted micromolding^31^. All these methods are based on the polymeric stamp, and are largely diffused in many laboratories since they are simple and do not require any sophisticated equipment.

## Results

**Figure 1:**
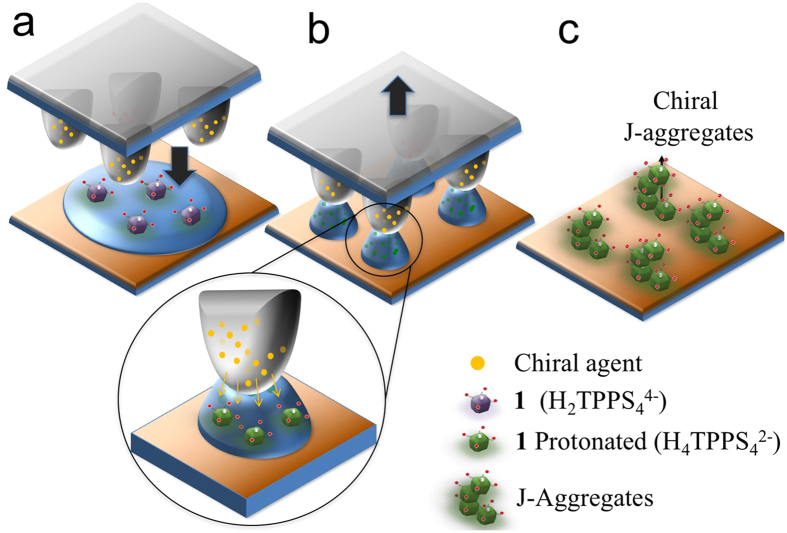
Scheme of the process. (**a**) A stamp pre-loaded with tartaric acid was placed over a film of **1** water solution spread onto the substrate. (**b**) On solvent evaporation, the solution stays pinned only to the protrusions, while the region in between the protrusions remains free of solution. During this stage, PDMS stamp releases the chiral agent (tartaric acid) due to osmotic effect. (**c**) When solution becomes supersaturated, aggregates form in the box shaped between the stamp protrusions.

**Figure 2:**
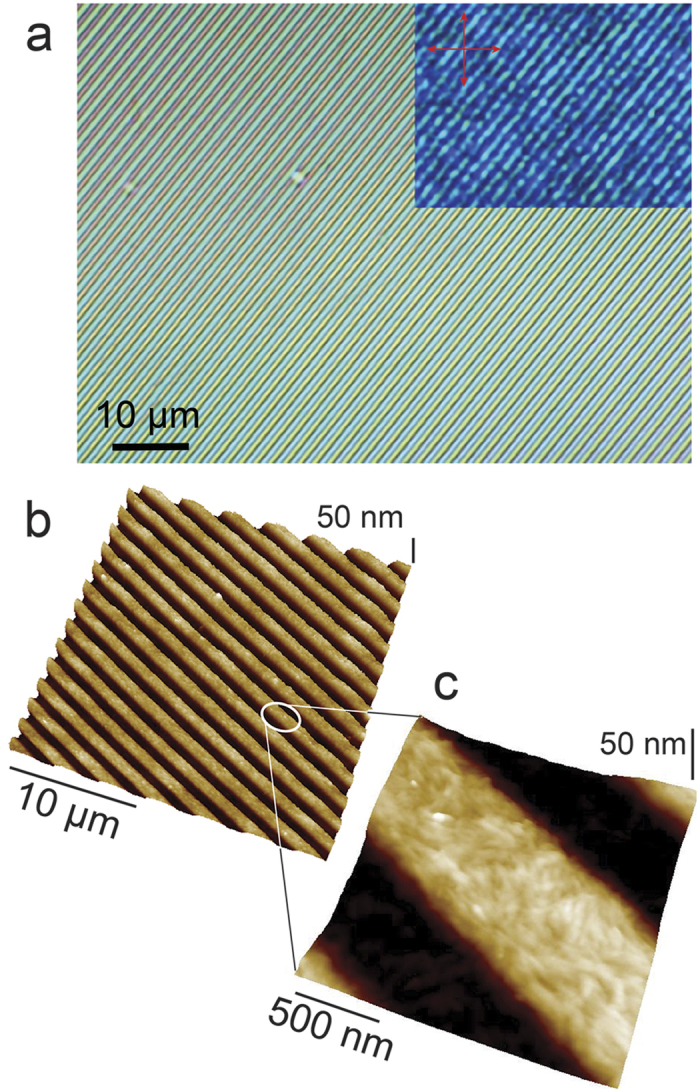
(**a**) Optical micrograph of printed stripes in bright filed and with crossed polarizers (inset). (**b**) Corresponding AFM topography at the micro- (**b**) and nano-metric (**c**) scale.

In our experiments (see a schematic sketch in Fig. 1), a polymeric stamp made of polydimethylsiloxane (PDMS) is loaded with a chiral agent by exploiting the PDMS property of swelling in ethanol. The stamp is immersed in a solution of (L or D) tartaric acid in ethanol for at least 12 hours, so allowing the alcohol to swell the polymer and the chiral agent to diffuse inside the stamp motif. Due to the low swelling ratio of PDMS in ethanol (<1.04)^32^, this solvent does not significantly alter the stamp features, preventing possible deformation during the printing process. The functionalized stamp is then placed in contact with an aqueous solution of the free base porphyrin, previously spread on a silicon or glass substrate (Fig. 1a). The menisci forms under the stamp protrusions by capillary forces. Due to osmotic effect, the PDMS stamp releases part of the tartaric acid which promotes the initial conversion of the free-base porphyrin in its diacid form (see the inset in Fig. 2b). As water evaporates, the solution remains pinned to the protrusions, dewetting^33^ the region between the protrusions free of solution (Fig. 2b). As the solution reaches supersaturation the aggregates form into the “confined box” imposed by the stamp motifs.

The confinement of the system provides suitable conditions both to drive porphyrin self-assembly and to induce chirality on the final aggregates. The quantity of released chiral agent can be tailored by adjusting the concentration used during the loading of the stamp. In optimized conditions, we demonstrated that loading the stamp with a 500 mM solution of tartaric acid and using a swelling time of 12 hours suffices to induce chiral aggregation in printed structures. In order to have optical accessible structures and to prevent the possible effect of dimensionality at the nanoscale, we used a stamp with micrometric motifs consisting of parallel lines 1 μm width and 1.5 μm spaced. However the printing process has been demonstrated to be efficient both at lower resolution (e.g. printing macro-structures) and at higher resolution (e.g. printing nano-structures)^24^. Printed samples were characterized by means of polarized optical microscopy (POM), atomic force microscopy (AFM), UV/Vis, fluorescence emission, and the chirality by circular dichroism (CD) spectroscopy.

## Discussion

Figure 2a shows an optical micrograph of J-aggregates printed in parallel micrometric stripes. The thickness of printed stripes can be tailored in a range of 20–200** **nm depending on the initial concentration of **1**. The structures thicker than 30 nm (indicative value) exhibit the green colour typical of these J-aggregates. Moreover, when observed by POM with crossed polarizers (inset of Fig. 2a), they exhibit a moderate birefringence, without any preferential directions of extinguishment. This behaviour suggests a polycrystalline structure in the printed stripes. The colour tends to turns to violet in the zones thinner than 30 nm. This difference can be ascribed to the smaller thickness, even if we cannot exclude the presence of amorphous aggregates of **1**.

The morphology of printed structures was investigated by AFM (Fig. 2b). Although the stamp imposes the mean morphology of stripes at the micrometric scale, a detailed investigation at nanoscale (Fig. 2c) reveals that the printed stripes are formed by small, rod-like nano-aggregates, whose length spans from 100 nm up to 400 nm with an aspect ratio ranging between 1:4 and 1:5. Our findings are in agreement with several structural investigations on these J-aggregates, which have been characterized as hollow nanotubes eventually collapsing in bilayered nanoribbons due to solvent evaporation^15^.

**Figure 3:**
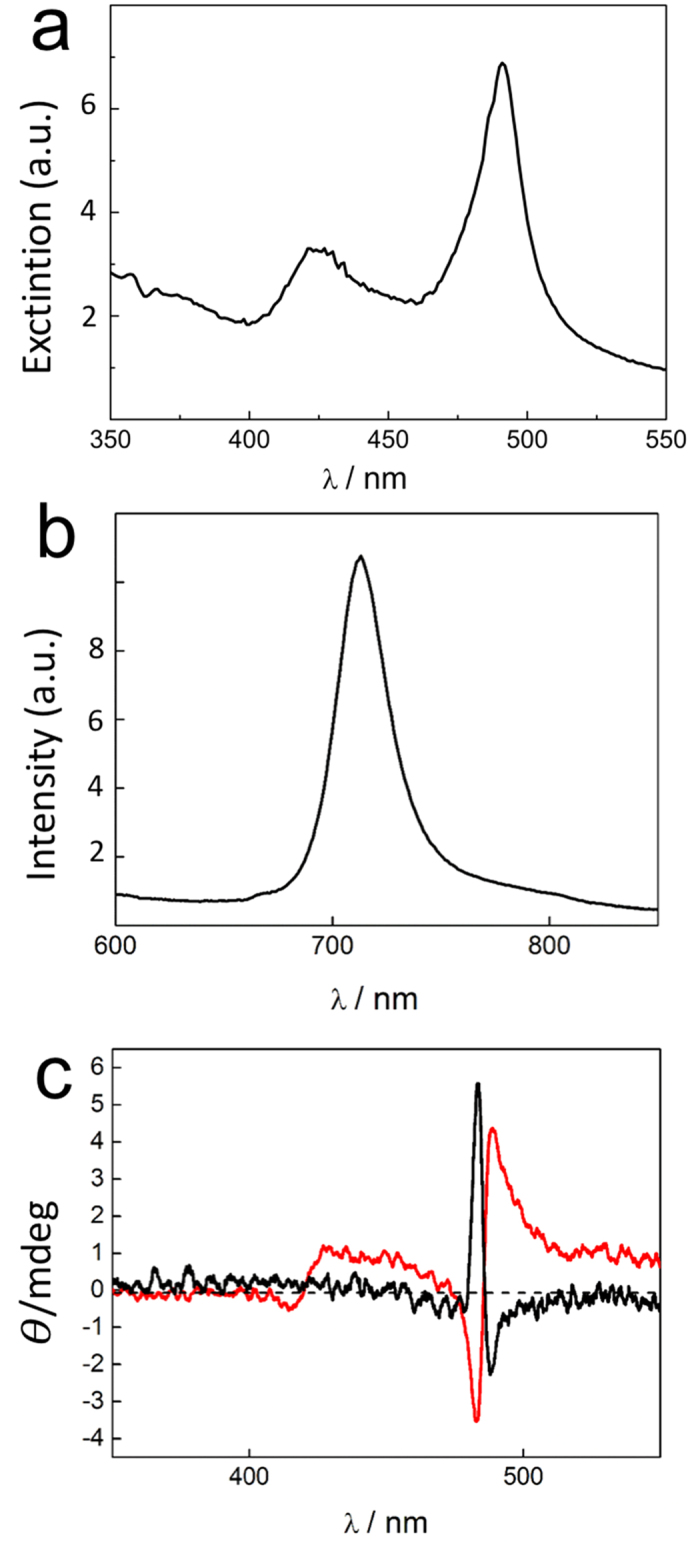
(**a**) UV/Vis extinction, (**b**) fluorescence emission spectrum (λ_exc_ = 490 nm) and (**c**) CD spectra of J-aggregates of opposite handedness deposited on glass surface by LCW using PDMS stamp functionalized with L (black curve) or D (red curve) tartaric acid.

Despite a slight tendency of the nano-aggregates to orient along the stripes, no evident order was observed inside them. No relevant quantity of material was detected between the stripes by POM or AFM. The optical/chiral properties of printed structure were investigated by a combination of UV/Vis extinction, fluorescence emission and CD spectroscopy (Fig. 3).

The UV/Vis extinction spectrum displays a J-band cantered at 490 nm, accompanied by the H-band at 422 nm typical of **1** aggregates (Fig. 3a). The nanoaggregates, excited on their J-band, exhibit a moderate fluorescence emission with a band cantered at 710 nm very similar to that already reported in bulk solutions (Fig. 3b)^34^. Typical CD spectra obtained by using stamps functionalized with D or L-tartaric acid evidence a clear bisegnate Cotton effect at the J-band absorption region (Fig. 3c). This observed profile is due to excitonic coupling and it is in agreement with previous observations on J-aggregates in bulk solutions^19^. Even if not totally symmetric, the two spectra (D or L) are almost mirror images, as expected for aggregates of opposite handedness. The experimental findings prove the effective chiral J-aggregation under lithographic control. No evidence of chirality was observed by CD in printed structure without the presence of the chiral agent on the stamp.

The dissymmetry factor *g* (g = ΔA/A, where A is the absorbance of the sample and, ΔA = AL − AR, with AL and AR the absorbance of left- and right-handed circular polarized light, respectively) is a good indicator of the chiroptical response and a value of 0.004 obtained for the printed structures is very similar to that already reported in literature for aggregates in solution^13^,^20^ or in microemulsion water pools^19^. A comparison between the aggregates size and the CD signals obtained in printed structures and in solution suggests very similar structures and that each nanoaggregate is formed by a single domain. Further investigations are currently underway to elucidate this issue.

Within a variation of CD signal of a few millidegrees, the process is highly reproducible, however, it must be noted that the protocol of fabrication is very important in order to obtain a transfer of chirality strictly related to the bulk solutions. As observed in solution, changing the order of mixing and/or the relative concentration of reagents leads to inverted or null CD signals. Indeed, if the chiral agent is added to the porphyrin starting solution, before applying the stamp (i.e. applying conventional LCW procedure for patterning), we obtained printed structures with inverted CD spectra (see SI). These parameters give our method the extraordinary possibility to tailor the chirality at solid state by processing, exploiting the know-how developed for aggregation in solution.

## Conclusions

In conclusion, we propose an important advance using a soft-lithographic approach to patterning supramolecular aggregates with the local spatial control of chirality. More specifically, we demonstrate the possibility to print nanometric structures exhibiting a programmed chirality induced by specifically functionalized stamps in a confined environment. We used standard established methods already proven as efficient to integrate functional nanostructures in several kinds of devices and model materials as a proof of concept. It is worth noting that the proposed approach is quite general and can be potentially extended to many other systems processable by soft-lithography. The future challenge in exploiting this method will now be to control the chiral self-assembly depending upon the nature of the chiral inducing agent loaded in each stamp motif. A qualitative and quantitative control on the patterned surface represents an outstanding perspective to improve this viable and cheap method to achieves well-defined chiral materials directly exploitable for many technological applications.

## Methods

### Samples in solution

10 μL of 2.0 mM aqueous solution (ultrapure-grade quality) of **1** (H2TPPS4^4−^, (Aldrich & Co., sodium salt) was acidified with 10 μL of EtOH solution of D or L tartaric acid (500 mM) up to a final concentration of 1.0 mM. Experimental conditions for spectroscopic characterization of porphyrin monomer and J-aggregates in solution were: [**1**] = 1 mM, [tartaric acid] = 250 mM, optical path-length 0.001 cm, exc = 490 nm, T = 298 K.

### Stamps and printing

The elastomeric polydimethylsiloxane (PDMS, Sylgard 184 Down Corning) stamps were prepared by replica moulding of a Compact Disk support that acts as a structured master. PDMS curing was carried out for 6 hours at 60 °C. Once cured, the PDMS stamp was washed in pure ethanol for one hour. The stamp motifs consist of parallel lines with 1.5 μm periodicity, 800 nm width at half height and 200 nm tick. The distance between the stamp and the surfaces was regulated by some spacers, 100 nm tick, present in the stamp, or prefabricated on the substrates. The stamps were immersed in ethanol solution of high purity D (Aldrich, 99%), or L (Aldrich, 99.5%) tartaric acid (500 mM) at room temperature for at least 12 hrs. After this time the stamps were removed from ethanol, washed with fresh pure ethanol several times and dried under nitrogen. The stamps embedded with the opportune chiral agent were used for LCW combined with release of the chiral agent^35^. The substrates consist of a 10 × 10 mm^2^ piece of silicon wafer with 200 nm of SiO_2_ or a commercial cover glass for optical microscopy. They were cleaned by sonication for 2 min in electronic-grade water, 2 min in acetone (Aldrich, chromatography grade), and eventually 2 min in 2-propanol (Aldrich, spectroscopic grade). The PDMS stamp was placed in contact with a liquid film of solution of **1** (20 μL/cm^2^) in pure water, previously spread onto the substrate. After 24 h it was removed.

### Characterization

UV/Vis absorption spectra were recorded on a Hewlett-Packard mod. 8453 diode array spectrophotometer. CD spectra were obtained on a JASCO J-720 spectropolarimeter, equipped with a 150 W xenon lamp. To quantify the sign and magnitude of the supramolecular chirality we used the dissymmetry factor Δg = g(489 nm) − g(483 nm), where g is the ratio of the CD to the conventional absorption Optical micrographs were recorded with a Nikon i-80 microscope equipped with cross polarizers. AFM imaging was performed on a Multimode 8 microscope r(Bruker, USA). Samples were scanned at 1.1 Hz/line in PeakForce mode using Scanasyst-Air probes (Bruker, USA) in air, using an applied force of 2.5 nN. Image levelling and surface analysis were performed by Gwyddion 2.37 (http://gwyddion.net/). Stripes thicknesses were measured from topographic heights distribution.

## Additional Information

**How to cite this article:** Castriciano, M. A. *et al*. Spatial control of chirality in supramolecular aggregates. *Sci. Rep.* **7**, 44094; doi: 10.1038/srep44094 (2017).

**Publisher's note:** Springer Nature remains neutral with regard to jurisdictional claims in published maps and institutional affiliations.

## Supplementary Material

Supplementary Information
