# Supplementary material for: Spatial control of chirality in supramolecular aggregates
Source: Sci Rep. 2017 Mar 9;7:44094. doi: 10.1038/srep44094 (PMC5343480; doi:10.1038/srep44094)
Supplement: Supplementary Information [file srep44094-s1.pdf]

Spatial control of chirality in supramolecular aggregates

Maria A. Castriciano,[a*] Denis Gentili,[a] Andrea Romeo,[a,b] Massimiliano Cavallini,[a*] Luigi Monsù Scolaro[a,b]

Supporting Informations

Characterization in solution

Figure S1 shows the structure of the diacid $H_4TPPS_4^{2-}$ and a schematic drawing of its J-aggregate, together with the corresponding spectral properties in terms of UV/Vis absorption, fluorescence emission and circular dichroism (in the presence of D or L-tartaric acid). For comparison the UV/Vis spectra of the parent free base porphyrin and the diacid form are also displayed.

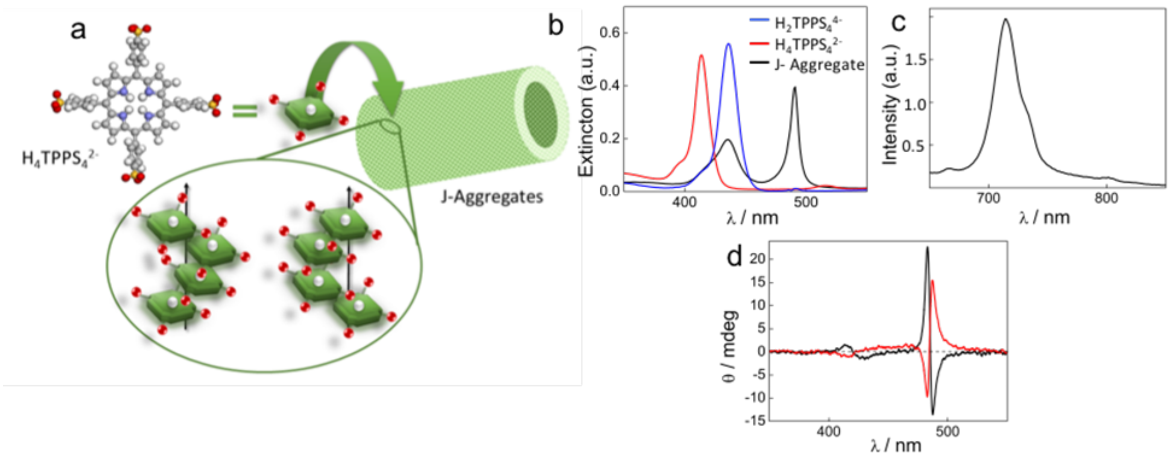

Figure S1. a) Chemical structure of the achiral diacid porphyrin $H_4TPPS_4^{2-}$ schematically represented by the green platelet. The monomers self-assemble into chiral J-aggregates in presence of chiral acids. b) UV/vis spectrum of $H_2TPPS_4^{4-}$ free base (red line), $H_4TPPS_4^{2-}$ diacid (blue line) and its J-aggregates (black line). J-aggregates exhibit an absorption at 490 nm, red-shifted in comparison to the diacid monomer B-band at 434 nm. c) fluorescence emission spectrum of J-aggregates (black line) exhibit a band centered at 715 nm. d) CD spectra of J-aggregates of opposite handedness obtained in solution by addition of L (black line) or D tartaric acid (red line). The dissymmetry factor *g* ($\Delta A/A$) = 2 x 10^{-3}.

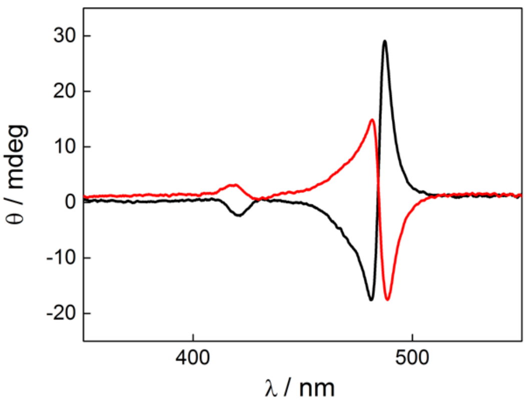

Figure S2. CD spectra of J-aggregates of opposite handedness obtained by adding L (black curve) or D (red curve) tartaric acid and deposited on glass surface by LCW method using unfunctionalized PDMS stamp
